# Shiga Toxins as Antitumor Tools

**DOI:** 10.3390/toxins13100690

**Published:** 2021-09-28

**Authors:** Aude Robert, Joëlle Wiels

**Affiliations:** 1 INSERM UMR1279, Institut Gustave Roussy, Université Paris-Saclay, 94805 Villejuif, France; 2 CNRS UMR9018, Institut Gustave Roussy, Université Paris-Saclay, 94805 Villejuif, France

**Keywords:** Shiga toxins, Gb3/CD77 glycosphingolipid, GL-Lect, signaling pathways, cell death, biomedical applications

## Abstract

Shiga toxins (Stxs), also known as Shiga-like toxins (SLT) or verotoxins (VT), constitute a family of structurally and functionally related cytotoxic proteins produced by the enteric pathogens *Shigella dysenteriae* type 1 and Stx-producing *Escherichia coli* (STEC). Infection with these bacteria causes bloody diarrhea and other pathological manifestations that can lead to HUS (hemolytic and uremic syndrome). At the cellular level, Stxs bind to the cellular receptor Gb3 and inhibit protein synthesis by removing an adenine from the 28S rRNA. This triggers multiple cellular signaling pathways, including the ribotoxic stress response (RSR), unfolded protein response (UPR), autophagy and apoptosis. Stxs cause several pathologies of major public health concern, but their specific targeting of host cells and efficient delivery to the cytosol could potentially be exploited for biomedical purposes. Moreover, high levels of expression have been reported for the Stxs receptor, Gb3/CD77, in Burkitt’s lymphoma (BL) cells and on various types of solid tumors. These properties have led to many attempts to develop Stxs as tools for biomedical applications, such as cancer treatment or imaging, and several engineered Stxs are currently being tested. We provide here an overview of these studies.

## 1. Introduction

Shiga toxins are named after Dr. Kiyoshi Shiga, the Japanese bacteriologist who first described the dysentery bacillus, *Shigella dysenteriae*, in 1897 [1]. Shiga toxin (Stx) is produced by *Shigella dysenteriae*, but similar toxins are also produced by strains of *Escherichia coli* belonging to the “Shiga toxin-producing *E. coli* (STEC)” group. These toxins are grouped into two types—Stx1 and Stx2—each of which has several variants (Stx1, Stx1c, Stx1d, Stx2, Stx2c, Stx2d, Stx2e, Stx2f, Stx2g, Stx2h, Stx2i and the recently identified Stx2k [2]). Molecular characterization has revealed that Stx1 is closely related to Stx (only one amino-acid different, in the A-subunit), whereas Stx2 has an amino-acid sequence only 56% identical to that of Stx. The variants display between 84 and 99% sequence identity to Stx1 or Stx2. The toxins produced by STEC have also been reported to be able to kill Vero cells [3], which has led to them often being referred to Vero cytotoxins or verotoxins (VT). Shiga toxin-producing bacteria may produce a single type of toxin, or a combination of variants of one or both types [4]. 

The pathogenesis of *Shigella* and STEC infections requires ingestion of the bacterium via contaminated food or water. Infection causes bloody diarrhea and other pathological manifestations that can lead to HUS (hemolytic and uremic syndrome), which is characterized by acute renal failure and thrombocytopenia. *Shigella dysenteriae* serotype 1 and STEC infections are a major public health concern worldwide, with acute infections in more than 2.8 million patients and almost 4000 HUS cases per year, mostly in the young and elderly. HUS occurs in about 10% of STEC-infected children and is the leading cause of pediatric renal failure; about 5–30% of patients suffer from chronic renal insufficiency, hypertension, or neurological deficits following the resolution of HUS [5]. There are currently no approved treatments for preventing or treating HUS caused by STEC [6]. 

The members of the Stx family are AB5 proteins composed of a single catalytic 32 kDa subunit A bound noncovalently to a pentamer of B subunits (7.7 kDa). This B subunit pentamer binds to the globotriaosylceramide (Gb3, Galα1–4Galβ1–4Glcβ1–1Cer) neutral glycosphingolipid (GSL) receptor expressed on the epithelial and endothelial cells of the intestine and kidney. Gb3 is also present on germinal center B lymphocytes and Burkitt’s lymphoma (BL) cells, on which it is called the CD77 antigen [7,8]. Moreover, Gb3 is the rare Pk blood group antigen [9]. The structure of the fatty-acid part of Gb3 (chain length, degree of unsaturation and hydroxylation) embedded in the plasma membrane greatly affects the conformation of the carbohydrate moiety exposed on the cell surface, playing a major role in the function of Gb3 as the receptor for Stxs [10]. Conversely, the high affinity of Stxs for Gb3 probably results from the presence of at least two, and up to three Gb3 binding sites per subunit B monomer [11] which means that one Stx can bind up to fifteen Gb3. It must also be mentioned that the variant Stx2e, which causes the edema disease in pig, binds preferentially to another GSL of the globoseries called globotetraosylceramide (Gb4) obtained by adding a N-acetylgalactosamine to Gb3 [12]. 

After binding to their receptor, Stxs undergo endocytosis by various mechanisms. Clathrin-dependent uptake was described years ago, and was subsequently shown to be mediated by the Stxs-induced activation of Src and Syc, which phosphorylate clathrin and increases the number of clathrin-coated pits [13]). 

Clathrin-coated pit-independent mechanisms involving Gb3 present in the lipid raft structures have also been reported. Lipid rafts are stable membrane subcompartments (of nanometer size) enriched in glycosphingolipids, cholesterol and specific proteins [14,15]. The presence of Gb3 in lipid rafts is correlated with cell sensitivity to the toxin [16]. Caveolae, flask-shaped invaginations of the plasma membrane with a structure similar to that of lipid rafts but consisting principally of caveolin and cavin proteins, are also involved in the internalization of Stxs, notably through specific membrane tubulation [17]. 

Finally, a broader mechanism, known as the glycolipid–lectin (GL-Lect) hypothesis, has also been described for Stxs internalization. This model is based on the demonstration that the binding of Stxs, via the three binding sites of the B subunit, to several Gb3 molecules located in lipid rafts induces a reorganization of the plasma membrane, leading to a strong clustering of the Gb3/Stxs complexes eventually resulting in tubular membrane invaginations [18,19,20]. Interestingly, these studies have also shown that the internalization of some galectins—a class of lectins that bind to galactose- and N-acetylactosamine-containing motifs—requires a similar mechanism. For example, galectin-3 (or 4), once bound to some of the glycosylated partners with which it interacts (CD44, β1-integrin), oligomerizes and induces membrane bending and the formation of tubular endocytic pits dependent on the presence of GSL in the membrane [21]. The tubular invaginations then detach from the membranes in an actin-dependent manner to form clathrin-independent carriers (CLICs) [22], which drive the uptake of their components into cells. Many questions about the fine-tuning of this GL-Lect mechanism remain unanswered, but it has been suggested that it could constitute a major alternative to the cytosolic clathrin machinery for endocytosis [23].

**Figure 1:**
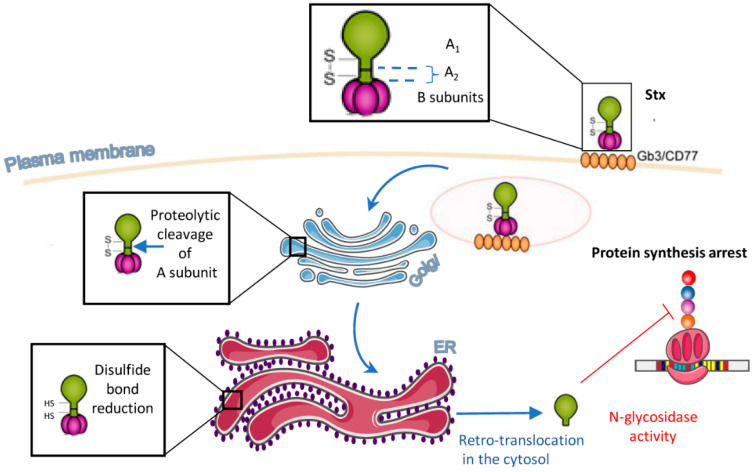
Overview of the intracellular trafficking of Stxs. Stx binds to Gb3 on the cell surface and is internalized by various endocytosis mechanisms. Stx is then transported to the Golgi apparatus, where the A-subunit is cleaved but remains attached to the B-subunit via a disulfide bond between A1 and A2. Stx then undergoes retrograde transport to the ER. There, the disulfide bond is reduced, leading to retrotranslocation of the enzymatic A1 fragment to the cytosol. Finally, StxA1 inhibits protein synthesis by cleaving an adenine residue from the 28S RNA of the 60S ribosomal subunit.

It is currently thought that the fraction of Stxs taken up by these different mechanisms varies between cell types. However, regardless of the endocytosis pathway used, the holotoxin is retrogradely transported to the Golgi apparatus and endoplamic reticulum (ER). During this retrograde transport, the A subunit is cleaved by furin or calpain into two fragments: the enzymatically active 27 kDa A1 and the small (6 kDa) A2, which remain linked by a disulfide bridge [24]. In the ER, the disulfide bond is reduced, allowing the translocation of StxA1 to the cytoplasm, with A2 and the B-subunit remaining in the ER. In the cytoplasm, StxA1 exerts its RNA glycosidase activity and removes an adenine from the 28S rRNA. This inhibits the catalytic activity of the 60S ribosomal subunit, ultimately blocking protein synthesis (Figure 1).

There are currently no vaccines for preventing the diseases caused by Stxs and, despite improvements in recent years, there is still no globally effective treatment for these conditions [25]. Additional in-depth studies are, therefore, required to shed light on the mechanisms of action of these toxins. The studies performed to date have focused on toxin-induced alterations to ribosomal structure and activity initiating cell stress pathways—the ribotoxic stress response (RSR) and the unfolded protein response (UPR)—and the triggering of other signaling pathways, such as autophagy and apoptosis, by Stxs. The activation of these signaling cascades induces cell death in most cells. 

Moreover, high levels of Gb3 have been reported in various cancers, including B-cell lymphomas and some solid tumors, such as breast, testicular and ovarian carcinomas [26,27,28]. Several engineered Stxs have thus been developed and are currently being tested as potential anticancer agents. In parallel, the potential of the non-active binding subunit, StxB, as a delivery tool for the treatment or imaging of Gb3-positive tumors is also being explored. We provide an overview of these investigations in this review.

## 2. Multiple Stxs-Triggered Signaling Pathways 

**Figure 2:**
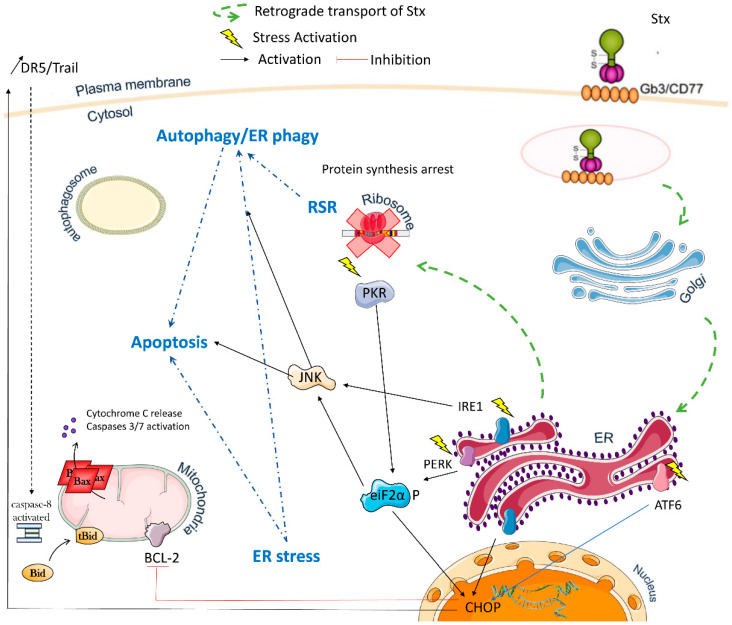
Stxs triggers multiple signaling pathways. This figure summarizes the different pathways observed in different cell types.

Stx triggers multiple cellular signals that are detailed below and summarized in Figure 2.

### 2.1. Ribotoxic Stress 

The RSR involves an evolutionarily conserved function of the ribosome, which can sense stress in highly conserved regions of the 28S ribosomal RNA (28S rRNA), leading to the induction of a cellular response involving the activation of mitogen-activated protein kinases (MAPKs) [29,30]. The link between MAPK activation and changes in ribosomal function is not yet fully understood. The RSR can be induced by Stxs, but also by other toxins, such as ricin, and it results from the depurination of the 28S RNA [31]. However, the RSR is a specific response, in that not all inhibitors of protein synthesis can induce it. For example, cycloheximide or puromycin, two potent protein synthesis inhibitors, cannot trigger the RSR. The RSR is thought to induce the expression of proinflammatory cytokines, potentially contributing to the severe outcomes observed in some cases of STEC infection. The damage to the 28S rRNA triggered by the catalytic A subunit of Stxs induces the activation of protein kinase R (PKR), a serine/threonine kinase that phosphorylates the translation factor eIF2a leading to its inactivation and the inhibition of protein synthesis [32]. It should be noted that the UPR, another cellular stress response, also involves the phosphorylation of eIF2a. This initiates a signaling cascade leading to activation of the classical p38 MAPK, ERK (extracellular-signaling regulated kinase) and JNK (c-jun N-terminal kinase). However, the roles of these kinases seem to be cell type-dependent. For example, p38 MAPK and JNK are involved in the apoptosis of epithelial cells; their inhibition prevents cell death in these cells, but not in monocytes. By contrast, in monocytes, the inhibition of p38 and ERK results in a decrease in cytokine production [33,34,35,36,37]. On the other hand, we have shown that the Ramos BL cell line expresses activated p38 MAPK in basal conditions, and that Stx1 treatment induces no further activation of this kinase. Indeed, p38 MAPK inhibitors may even sensitize these lymphoma cells to apoptosis induced by Stx1, suggesting that p38 MAPK promotes survival and protects cells against Stx1-induced apoptosis [38].

### 2.2. ER Stress

Many studies have shown that Stxs can trigger endoplasmic reticulum (ER) stress. The ER is a eukaryotic organelle with crucial functions, serving as the primary site for correct protein folding and a major intracellular calcium store. These functions are controlled by three major proteins, the “ER stress sensors”: IRE1 (inositol-requiring enzyme 1), PERK (PKR-like ER protein kinase) and ATF6 (activating transcription factor 6). In normal conditions, these proteins are associated with the chaperone BIP (binding immunoglobulin protein, also known as GRP78). When unfolded proteins accumulate in the ER, UPR is triggered: the sensors are dissociated from BIP, allowing their activation [39], PERK and IRE1 are activated by autophosphorylation after homodimerization, whereas ATF6 is activated by proteolytic cleavage after translocation to the Golgi apparatus. The activation of ER membrane sensors results in the transient attenuation of overall protein translation concomitant with the transcriptional activation of genes encoding chaperones and proteins involved in the ER-associated protein degradation pathway (ERAD), which mediates the degradation of misfolded proteins. Prolonged UPR signaling (due to a failure to correct protein folding defects or to maintain Ca^2+^ homeostasis) can lead to apoptosis. In this case, CHOP (C/REB homologous protein, also known as GADD153) is the key transcription factor directly or indirectly regulating the expression of genes involved in apoptosis [40].

Differences between cell types in the ability of Stxs to activate the ER stress sensors have been observed. Indeed, in monocytic THP-1 and retinal pigment epithelial cells (ARPE-19), Stx-1 treatment induces the activation of all three sensors [33,41], whereas, in macrophage-like cells (differentiated THP-1 cells), IRE1 and PERK are activated, but ATF6 is not [42], in BL cells, IRE1 and ATF6 are activated, but the PERK/eiF2a/ATF4 pathway is not [43], and in HK-2 cells (immortalized human proximal tubule epithelial cells), only ATF6 is activated. Differences in the ability of Stx1 and Stx2 to activate these sensors have also been reported. For example, in HK-2 cells, Stx2 can activate the PERK and IRE1 pathways, but not ATF6 [44]. Interestingly, mutant toxins with no RNA glycosidase activity (Stx1A- or Stx2A-) can also induce the activation of ER sensors, either more transiently [33] or only some of them [41]. Finally, it does not seem to matter which pathway is activated, because prolonged signaling via PERK, IRE1 or ATF6 leads to the upregulation of CHOP, mostly resulting in cell death [33,41,42,43,44].

Indeed, CHOP appears to be upregulated after Stx treatment in all cell types, and this upregulation seems to be dependent on the enzymatic activity of the A subunit [33,42]. However, in some cases, CHOP accumulation does not lead to apoptosis: CHOP silencing has no effect on cell death induced by Stxs in Ramos BL cells and THP-1 monocytic cells [43,45]. On the contrary, macrophage-like THP-1 cells are partially protected against cell death. This led Lee et al. to suggest that the different ER stress signaling pathways activated in undifferentiated monocytic THP-1 and macrophage-like THP-1 cells might account for the observed differences in sensitivity to Stx [46]. In BL cells too, ER stress may enhance Stx1-induced apoptosis through CHOP or play a protective role through ER-phagy, depending on the cell line [43]. It has also been shown that Stx2 induces renal ER stress and apoptosis in murine models of Stx2-induced kidney injury, but that the inhibition of these processes has no effect on survival outcome [47]. All these findings suggest that the CHOP pathway is not the principal pathway of apoptosis induction and that survival pathways can be triggered following Stx infection. Indeed, ER stress, including the PERK/ATF4, IRE1 and ATF6 pathways, or Ca^2+^ modulation can induce cell apoptosis, but may also cause autophagy.

### 2.3. Autophagy

Macroautophagy (hereafter referred to as autophagy) is a fundamental process that maintains cell homeostasis by enabling cells to degrade and recycle their cytosolic components. Autophagy has been reported to protect cells from death, but, under certain circumstances, it may also contribute to cell death.

Several groups have used autophagy inhibitors to demonstrate that autophagy may be necessary for Stxs to induce apoptosis in Vero [48], HK-2 [46] and CaCo2 cells [49] cells. Moreover, Tang et al. demonstrated that Stx2 induced autophagic cell death through ER stress in CaCo2 intestinal epithelial cells [49]. Lee et al. showed that autophagy was induced in cells (human monocyte-derived macrophages) that survived Stxs treatment, but also in cells (undifferentiated THP-1 and HK-2 cells) undergoing apoptosis. They suggested that calpain- and caspase-mediated cleavages of ATG5 and BECLIN convert a prosurvival autophagic response into an apoptotic response in toxin-sensitive cells. They also showed that toxin enzymatic activity was not required for the induction of autophagy [46]. By contrast, in the Ramos BL cell line, autophagy inhibitors increase Stx1-induced apoptosis, suggesting that autophagy may protect these cells against Stx1 toxicity, but not sufficiently to prevent cell death [43]. These findings again indicate that despite the activation of common pathways by Stxs, the effects observed depend on cell type. Moreover, Stx-induced autophagy seems to be closely related to ER stress.

### 2.4. Apoptosis

The induction of apoptosis by Stxs was first reported in 1986, when the morphological changes typical of this type of programmed cell death were observed in the intestinal epithelial cells of rabbits inoculated with STEC or prepared Stx [50,51]. The induction of apoptosis, as attested by morphological modifications or DNA fragmentation, has also been reported in various other cells treated in vitro with Stxs [52,53,54,55]. Many studies have since analyzed the molecular mechanisms of apoptosis triggered by Stxs. These studies have shown that Gb3 expression on the cell surface is essential, but not sufficient (as described above) to trigger apoptosis. They also implicated various pathways in different cell types, such as lymphoid, myeloid, epithelial and endothelial cells. The first results showed the activation of various tyrosine kinases (Yes, Lyn, Syk) soon after the binding of Stxs to Gb3 [56,57], but it rapidly became clear that the caspase cascade played a major role in Stxs-induced apoptosis. Indeed, it was shown that Stxs induced the activation of caspases-8 and -3 in THP1 monocytic cells [58], BL cells [59], HEp-2 epithelial cells [60], HeLa cells [61] and human microvascular endothelial cells (HMEC) [55].

The signaling pathway was then further analyzed in various cell lines. In BL cells, we showed that the apoptotic signaling cascade involved degradation of the caspase-8 inhibitory molecule c-FLIP_L_, the activation of caspase 8, which cleaves BID, and the activation and relocalization to the mitochondrial membrane of BAX, which, together with BAK, participates in mitochondrial outer membrane permeabilization (MOMP) leading to the release of CYT C and SMAC/DIABLO from the mitochondria into the cytosol. We also demonstrated the involvement of both full-length BID (FL-BID) and its truncated form (t-BID) in this process: FL-BID controls the changes in BAK and BAX conformation leading to their activation, and their homo-oligomerization, whereas t-BID controls BAK/BAX interactions. Both homo- and heterooligomers then act in coordination to optimize the release of CYT C and SMAC/DIABLO [38,43,62]. In monocytic THP-1 cells, apoptotic signaling also involves caspase-8 activation, inducing the cleavage of BID, a loss of mitochondrial membrane potential and the release of CYT C from the mitochondria. However, in this case, caspase 8 activation is due to ER stress and, more precisely, the activation of calpain, a Ca^++^-dependent cysteine protease that can cleave pro-caspase 8 [41].

Two anti-apoptotic members of the BCL-2 family, BCL-2 and MCL-1, also seem to play a role in Stxs-induced apoptosis. In sensitive THP-1 cells, Stx-1 induces a decrease in BCL-2 levels, whereas a similar treatment results in an increase in BCL-2 levels in resistant cells; these differences reflect differences in the phosphorylation of BCL-2 [41,42]. However, it has also been reported that the transfection of BL cells with BCL-2 provides no protection against Stx-1-induced apoptosis [63]. Conversely, a decrease in MCL-1 levels, which is associated with the onset of apoptosis, was reported in HMEC cells treated with Stx1 and Stx2 [64], and a decrease in the levels of MCL-1 and cFLIP, which could trigger the activation of caspase 9 and apoptosis, was described in Gb3-positive U937 cells [65].

Finally, it should also be pointed out that most of the studies on Stxs-induced apoptosis were performed with the holotoxins. However, some teams have also tested the effect of cross-linking Gb3 with Stxs B subunits or specific antibodies. Some of these studies reported that anti-Gb3 antibodies and StxB trigger apoptosis (albeit by mechanisms different from those observed with holotoxins) [52,56,62,63,66], whereas others showed that StxB could not induce apoptosis [58,61,67,68]. One reason for this discrepancy may be the use of different cell types in these experiments.

## 3. Biomedical Applications of Shiga Toxins

### 3.1. Holotoxins as Antitumor Tools

Two particular properties of Stxs—the specific targeting of Gb3-positive cells and efficient delivery to the cytosol—are potentially useful for biomedical applications. Furthermore, Stxs are resistant to hostile conditions, both within cells and in the extracellular environment (they are stable at extreme pH and in the presence of proteases) and can cross tissue barriers, facilitating their distribution within the organism. Stxs have also naturally evolved to enter the cytosol of host cells by circumventing endosomal degradation. Whereas drugs conjugated to antibodies enter cells via the endosomal-lysosomal pathway, drugs conjugated to Stxs enter cells via the retrograde transport route, enabling them to escape lysosomal degradation. For all these reasons, considerable efforts have been made in recent years to develop the use of holotoxin or the purified A and B subunits for medical applications in the context of various diseases.

By contrast, Gb3, the cellular receptor of Stxs, has a restricted profile of tissue expression in normal cells [7] but is overexpressed on many tumors, including BL, glioma, testicular, ovarian and breast tumors, pancreas and colon cancers [26,28,69,70,71,72,73,74]. Moreover, Gb3 expression increases in metastatic breast and ovarian cancers [75] and Gb3 is expressed in the invading blood vessels of the tumors [76,77]. Cisplatin treatment has been shown to upregulate Gb3 expression, suggesting a possible link between Gb3 expression and chemoresistance [78].

The binding of Stx, via Gb3, to cells of both the endothelial and tumor subsets suggests that Stxs may have both antineoplastic and antiangiogenic potential. As described above, Stx induces apoptosis in various cancer cell lines in vitro in an extremely efficient manner (theoretically, one toxin molecule is enough to kill a cell). This has led to pioneering experiments with the holotoxin. A single intratumor injection of Stx has been shown to increase survival in nude mice xenografted with various tumors: human astrocytoma, malignant meningioma, or renal tumors [79,80,81].

However, the use of the holotoxin is limited by its considerable toxicity, as it can damage endothelial cells and cause hemolytic and uremic syndrome (HUS), as observed in patients with STEC infections [82]. Its potential for testing in clinical trials is also limited due to its immunogenicity, which is largely due to the A subunit [83,84]. Interestingly, the prevalence of anti-Stx antibodies has been shown to be high in healthy population and this may reflect population immunity to systemic Stx-associated disease [85].

These potential drawbacks have led to modified toxins being preferred over holotoxins for applications in humans. For example, deimmunization is a new technology in which molecular techniques are used to identify and remove B- or T-cell epitopes, to prevent immunogenicity whilst maintaining the therapeutic potential of proteins. This technology has already been developed for *Pseudomonas* exotoxin and *Diphtheria* toxin [86] and is currently being applied to Stxs for the engineering of new types of therapeutic tools (see below). The A and B subunits can also be used separately, the first to kill cells directly by the enzymatic inactivation of ribosomes, and the second for its capacity to target cancer cells.

### 3.2. The Antibody-Coupled A Subunit for Cancer Treatment

A new concept for increasing the specificity of the effects of toxins was proposed in the 1970s: the linking of the enzymatic subunit of the toxin to a specific antibody (or to cytokines). The resulting chimeric molecules were named immunotoxins (ITs) and, in 1999, Ontak, an immunotoxin consisting of human IL2 conjugated with a truncated form of diphtheria toxin (DT), was the first to be approved by the US Food and Drug Administration (FDA) for the treatment of cutaneous T-cell lymphoma (CTCL). Many ITs have been engineered with monoclonal antibodies or cytokines as targeting agents and various types of toxins (cholera toxin, *Pseudomonas* exotoxin A, Shiga toxin and *Diphtheria* toxin) [87], but only three have been approved by the FDA. Two (Ontak and tagraxofusp) contain DT, whereas the third (moxetumomab) is based on *Pseudomonas* exotoxin A.

Concerning Stx, Molecular Templates, a biopharmaceutical company, recently focused on the development of what they call “Engineered Toxin Bodies” (ETBs) for Stx. These ETBs consist of a modified Stx1 catalytic subunit A genetically fused to a specific single-chain variable fragment (scFv). The first ETB produced, MT-3724, recognizes CD20-expressing cells and is currently in phase II trials for the treatment of DLBCL (diffuse large cell B lymphoma) as a single agent [88] or in combination with lenalidomide (NCT03645395) or gemcitabine/oxaliplatin (NCT03488251). According to the preliminary results, when used in combination with lenalidomide, it yielded complete responses in two of the seven patients tested, partial responses in three patients, stable disease in one patient and progressive disease observed in the final patient [89]. In monotherapy, no life-threatening toxicities have been reported, but minor adverse effects (fatigue and peripheral edema) have been observed. One serious adverse event (grade 2 capillary leak syndrome) was detected in a patient treated with MT-3724 in combination with lenalidomide and resulted in dose limitation. The development of anti-drug antibodies or neutralizing antibodies (mostly directed against StxA) was observed, but some subjects nevertheless displayed an improvement.

In second-generation ETBs (such as MT-5111 (targeting HER2) and TAK-169 (targeting CD38) [90]), the scFvs were fused to a genetically engineered deimmunized StxA, to decrease innate and adaptive immunogenicity. These compounds are currently being tested in a phase I study. First results obtained with MT-5111 showed that it specifically binds and kills HER2-expressing cells and still binds to cells in the presence of other HER2 targeting agents (trastuzumab, pertuzumab or T-DMI (trastuzumab coupled to a drug called emtansine)). Therefore, MT-5111 can potentially be combined with—or replace–these agents and constitute a new tool to overcome tumor resistance to other therapies targeting HER2 [91]. Intermediate results obtained in a phase I open-label dose escalation and expansion study (NCT04029922) in patients with advanced HER2+ solid tumors showed no dose limiting toxicities nor cardiotoxicity and that the maximal tolerated dose has not been reached, which is very encouraging for the future [92,93].

In the third generation of ETBs, the molecule has been engineered further, to deliver an antigen derived from cytomegalovirus (CMV) inside the tumor for presentation on the cell surface in complex with MHC class I molecules. This technology, called “antigen seeding”, allows CMV-reactive T cells to recognize and destroy tumor cells in addition to the ribosomal inactivation caused by StxA. A third-generation ETB targeting PD-L1 (MT-6402 is currently being tested and a phase I study in PD-1/PD-L1 antibody relapsed/refractory patients is expected to be initiated in 2021. In preclinical studies, MT-6402 specifically binds and kills both tumor and immune PD-L1 expressing cells through ribosomal inactivation and CMV specific immune response. One hypothesis is that MT-6402 could act on the tumor microenvironment (TME) where it destroys PD-L1-expressing immune cells thereby allowing immune recognition of tumors [94,95].

ETBs are in development for other targets including CTLA-4, SLAMF7 and CD45.

### 3.3. The B Subunit as a Delivery Tool

StxB is an ideal tool for targeting Gb3-expressing cells. It is a protein with low immunogenicity that can withstand the extreme pH conditions in the gastrointestinal tract and cross the intestinal barrier. These properties could be exploited for the in vivo delivery of contrast agents or fluorophores to tumors for imaging and for targeted anticancer treatments. Various delivery systems based on StxB have been developed: StxB-drug conjugates, StxB-functionalized liposome conjugates and StxB-fusion proteins.

#### 3.3.1. *In Vivo* Imaging

One of the most important challenges in oncology is the early and specific targeting of primary tumors and metastases. As StxB is non-toxic, it can be coupled to imaging agents, such as fluorescent dyes or contrast agents.

The specific targeting capacity of StxB was initially evaluated in digestive tumors. Confocal laser endoscopy showed that orally administered FITC-coupled StxB reached intestinal tumors in mice. PET imaging showed that the contrast reagent, [^18^F] fluoropyridine-based maleimide coupled to StxB and injected into the retro-orbital sinus of the control mice, was delivered to normal tissues in the urinary tract, kidney, spleen, lungs and liver, but that the abdominal region was not labeled. By contrast, in adenocarcinoma-bearing mice, [^18^F]-StxB was able to reach tumor sites in the digestive tract [96]. Viel et al. showed that fluorescent StxB (coupled to Cyanine 5 or 3) administered intravenously to nude mice with subcutaneous xenografts of human colorectal carcinoma cells accumulated in the Gb3-expressing tumor cells, but also in the epithelial cells of the neovasculature and in the monocytes and macrophages surrounding the xenografts: three types of cells known to express Gb3. After administration, the fluorescent STxB was slowly eliminated by renal excretion [97].

STxB has also been used to functionalize microbubbles for ultrasonography. Microbubbles are used as contrast agents but, because of their size, their biodistribution is limited to the blood after injection. Functionalized microbubbles can be targeted to blood vessels and to vascular endothelial cells if the target is present on the accessible surface. Gb3 is overexpressed in the neovasculature of tumors, and STxB-functionalized microbubbles are therefore able to bind specifically to cells expressing Gb3 in vitro and in vivo, suggesting that they could be used for the ultrasound imaging of angiogenesis [98].

These experiments, although promising, showed that StxB binding is not limited to tumor area, but also found in normal tissues, raising questions about how to distinguish between background and specific signals, particularly for small tumors. They also highlight the importance of studying the possible adverse effects on normal tissues of the use of StxB to deliver drugs.

#### 3.3.2. In-Tumor Targeting

Cancer cells may carry up to 10^8^ StxB binding sites, whereas antibodies typically have, at most, 10^6^ binding sites per target cell. StxB is, therefore, likely to be more efficient than antibodies as a vector for the tumor-specific targeting of cytotoxic drugs and could be a carrier of choice for targeting Gb3-positive cancers [99]. Moreover, StxB can deliver molecules with a low solubility [100]. StxB has, therefore, been coupled to various chemotherapy drugs, including doxorubicin (a DNA intercalating agent), auristatin F (MMAF; an antimitotic agent that inhibits tubulin polymerization), and SN38 (a drug that inhibits topoisomerase I). StxB–doxorubicin and StxB–MMAF have been shown to be highly selective for and potent against Gb3-positive HT29 cells, with IC_50_ values in the nanomolar range [101]. The cytotoxic effect of STxB-SN38 on pancreatic and gastric cancer cell lines is more than 100 times stronger than that of irinotecan, the SN38 prodrug used in clinical practice [102,103]. StxB has also been coupled to chlorin and glycoporphyrin, two photosensitizers that produce cytotoxic reactive oxygen species (ROS) when exposed to light at certain wavelengths. Such photodynamic therapies have been widely used for the treatment of cancers and non-neoplastic disorders. StxB slightly enhances the delivery of chlorin in Vero cells, and the conjugate is 10 times more efficient for photodynamic cell killing than chlorin itself [104]. Moreover, the photosensitizing activity of glycoporphyrin conjugated with StxB is five times higher than that of the parent molecule [105]. However, no other more recent studies of the use of photosensitizers coupled to StxB have been reported.

Interest in the StxB fusion protein has recently increased. Notably, Stx2B has been used to overcome the inability of small engineered proteins called monobodies to cross cellular membranes. Monobodies are small proteins engineered with a fibronectin type III domain (FN3) as a molecular scaffold, which specifically bind and inhibit various cytosolic oncoproteins. A chimeric toxin composed of Stx2B and the translocation domain of *Pseudomonas aerugina* exotoxin A fused to monobodies targeting the Lck tyrosine kinase has been shown to be delivered to cells and to inhibit the kinase signaling pathway [106]. Mohseni et al. recently evaluated the antitumor potential of StxB conjugated to the diphtheria toxin against breast cancer cell lines in vitro [107].

However, most of these studies on StxB-drug conjugates were performed in vitro, with the notable exception of studies on the N8A (MDM2 inhibitor)-StxB fusion protein, which was shown to suppress tumor growth in hepG2-xenografted mice following intraperitoneal injection every two days [108]. Batisse et al. also reported moderate therapeutic potency for the SN-38-StxB conjugate in a xenograft tumor model in mice, even at the maximum tolerated dose of STxB [99] suggesting that additional engineering of StxB conjugates is required to improve in vivo efficacy and that additional in vivo studies are essential, to evaluate the real benefit of conjugating drugs with StxB.

StxB has also been used in antigen presentation studies, to induce protective cell-mediated immunity to improve the clearance of certain tumors. StxB can deliver antigen directly to dendritic cells, which then express it on their surface, inducing a specific T-cell response. Furthermore, the vaccination of mice with StxB coupled to E7, a protein derived from HPV16, has been shown to inhibit the growth of E7-expressing tumors [109]. StxB is, thus, a potentially attractive and competitive carrier protein for cancer vaccines.

## 4. Conclusions

Natural selection has made Stxs one of the most efficient carriers for the delivery of toxic proteins to cells through binding to a specific receptor on the cell surface. Stxs can be used as anticancer agents in their native form or as engineered variants. However, the attachment of a molecule to StxB or StxA can alter the chemical and physical characteristics of the original Stx, introducing variability in terms of stability, immunogenicity and biodistribution, with potential impacts on the off-target toxicity and efficacy of the complex. In addition, StxB is rather small, and its coupling to large molecules may impair its binding to Gb3. Stability and pharmacokinetics are therefore of crucial importance in the development of engineered Stxs.

One important issue concerning the use of Stxs, with or without modification, is the economic and environmental feasibility of their production. For example, the use of Stxs as anticancer tools will require large-scale (g or kg amounts) production of the Stx-derived protein. In most studies, Stx, StxA or StxB are produced in *E. coli*, but the overexpressed or recombinant proteins accumulate within inclusion bodies in this system, potentially limiting the amount of material obtained and necessitating further processing, leading to additional losses. Furthermore, production systems of this type require the removal of any other endotoxins produced by the bacterium before use in humans. As an illustration of this, the clinical use of Ontak has been discontinued due to issues relating to production in a bacterial expression system in which purification is difficult.

ETBs appear to be the most promising applications based on Stxs to date. Recent advances in genetic engineering have paved the way for the development of immunotoxins capable of killing target cells in a highly specific manner with only minor side effects and low levels of immunogenicity. However, preclinical and clinical studies are required to evaluate their efficacy and safety.

## Data Availability

No new data were generated in this study. Data sharing is not applicable to this article.
